# Individualized Trimethoprim-Sulfamethoxazole Dosing in Non-HIV Patients with Pneumocystis Pneumonia: A Narrative Review of Current Evidence

**DOI:** 10.3390/jpm15070311

**Published:** 2025-07-14

**Authors:** Ilias E. Dimeas, George E. Dimeas, George E. Zakynthinos, Vasiliki Tsolaki

**Affiliations:** 1 Department of Respiratory Medicine, University Hospital of Larissa, Mezourlo, 41335 Larissa, Greece; 2 Intensive Care Unit, Faculty of Medicine, University Hospital of Larissa, University of Thessaly, 41335 Larissa, Greece; vasotsolaki@yahoo.com; 3 Department of Internal Medicine, General Hospital of Karditsa, 43100 Karditsa, Greece; gedim06@hotmail.com; 4 3rd Department of Cardiology, “Sotiria” Chest Diseases Hospital, Medical School, National and Kapodistrian University of Athens, 11527 Athens, Greece; gzakynthinos2@gmail.com

**Keywords:** *Pneumocystis jirovecii* pneumonia (PJP), trimethoprim-sulfamethoxazole (TMP-SMX), individualized dosing strategies, non-HIV immunocompromised patients, inflammatory response, personalized immunomodulation, immune reconstitution inflammatory syndrome, precision medicine in infectious diseases

## Abstract

**Background:** *Pneumocystis jirovecii* pneumonia (PJP) remains a serious threat to non-HIV immunocompromised patients, who often experience rapid disease progression, delayed diagnosis, and higher mortality. Standard treatment with high-dose trimethoprim-sulfamethoxazole (TMP-SMX) is based primarily on data from HIV-positive populations, despite differences in immune response and drug tolerability. **Objective:** This narrative review critically synthesizes the available evidence on lower-dose TMP-SMX strategies for PJP in non-HIV patients and explores the potential role of individualized dosing approaches to improve outcomes. **Findings:** Emerging retrospective data suggest that lower-dose regimens (<15 mg/kg/day) may provide similar survival outcomes with fewer adverse effects. The intense inflammatory response observed after treatment initiation in non-HIV patients, potentially exacerbated by high-dose therapy, may contribute to clinical deterioration. This raises the possibility that TMP-SMX dosing itself could influence immune-mediated lung injury. While adjunctive corticosteroids are frequently used to temper inflammation, their benefit remains uncertain. **Conclusions:** Existing data suggest that lower-dose TMP-SMX may be effective and better tolerated in some non-HIV patients with PJP. A personalized approach to dosing, informed by clinical and host-specific factors, represents a promising strategy to optimize outcomes and minimize harm. Future research should prioritize precision medicine frameworks and prospective evaluation of individualized dosing protocols.

## 1. Introduction

*Pneumocystis jirovecii* pneumonia (PJP) is a severe opportunistic infection primarily affecting immunocompromised individuals, such as those with malignancies, having undergone organ transplants, or on immunosuppressive therapies. Although historically associated with the Human Immunodeficiency Virus/Acquired Immune Deficiency Syndrome (HIV/AIDS) epidemic, PJP remains a significant concern in non-HIV immunocompromised patients. The pathophysiology of PJP begins with the attachment of Pneumocystis trophozoites to alveolar type I pneumocytes via adhesive proteins and glycoproteins. The fungus transitions from its trophic to cystic form, damaging the alveolar epithelium through basement membrane disruption. Importantly, much of the resulting lung injury is caused by the host’s inflammatory immune response, leading to impaired gas exchange and respiratory dysfunction [1].

In the setting of immune responses that resemble both infectious and autoimmune processes, the term inflammation broadly refers to the host’s immune-mediated reaction to Pneumocystis infection. This includes elevated markers such as c-reactive protein and erythrocyte sedimentation rate, radiologic signs like ARDS, and systemic manifestations such as multiorgan dysfunction. On a molecular level, inflammation may involve cytokine storms and inflammasome activation, contributing to lung injury [2]. Distinguishing this sterile immune response from true microbial sepsis is often challenging, and misinterpretation can lead to unnecessary antimicrobial escalation instead of considering immunomodulatory therapy [3].

Clinically, PJP in immunosuppressed non-HIV patients tends to be more aggressive than in HIV-infected individuals, often presenting with rapid-onset symptoms such as dyspnea, dry cough, and fever [1,4]. It is associated with higher mortality and accelerated disease progression, likely due to a heightened inflammatory response triggered after the initiation of antimicrobial therapy, in contrast to the comparatively milder immune reactions observed in HIV-related PJP [1,4]. As a result, immunocompromised non-HIV patients experience higher rates of respiratory failure and mortality, making prompt and effective treatment critical [5]. Despite advances in understanding the disease, treatment remains challenging due to the unique cholesterol composition of the Pneumocystis cell membrane, which renders it resistant to most antifungals. Trimethoprim-sulfamethoxazole (TMP-SMX) remains the cornerstone of therapy for PJP [6].

In light of these challenges, a one-size-fits-all approach to TMP-SMX dosing in non-HIV PJP appears increasingly inadequate. Immunosuppressed patients vary widely in factors that directly influence both therapeutic efficacy and toxicity. The lack of individualized dosing strategies guided by host-specific profiles raises the risk of immune dysregulation and hyperinflammation following treatment initiation, possibly as a consequence of the drug’s fungicidal effect.

This narrative review challenges the appropriateness of currently established TMP-SMX dosing in non-HIV patients with PJP and explores the overlap between infection-driven immune activation and autoimmune-like inflammatory responses. Specifically, it evaluates the available evidence for lower-dose TMP-SMX strategies, their potential impact on survival and adverse events, and the rationale for individualized dosing approaches. In doing so, it aims to contribute meaningfully to ongoing discussions in both infectious diseases and autoimmunity, while supporting a precision medicine framework that better aligns with the biological and clinical complexity of PJP.

## 2. Methods

This is a narrative review conducted in accordance with the SANRA (Scale for the Assessment of Narrative Review Articles) guidelines. A non-systematic literature search was performed using PubMed and Web of Science to identify relevant English-language publications from 1975 to 2025. Keywords included combinations of “*Pneumocystis jirovecii* pneumonia,” “trimethoprim-sulfamethoxazole,” “non-HIV immunocompromised,” “low-dose,” “individualized dosing,” and related terms. References were selected based on their clinical relevance and contribution to the evolving understanding of TMP-SMX dosing strategies in non-HIV patients. No structured inclusion or exclusion criteria, quality assessment tools, or PRISMA methodology were applied, as this is a narrative review and not a systematic review.

## 3. Trimethoprim-Sulfamethoxazole Dosing Studies in Immunocompromised Non-HIV PJP

High-quality randomized trials comparing TMP-SMX dosing regimens for PJP are lacking [7,8]. Current guidelines recommend a weight-based TMP-SMX dosage of 15–20 mg/kg/day of the trimethoprim component as the standard of care. This dosing recommendation for immunosuppressed non-HIV PJP was first established in a small pediatric observational study conducted in the 1970s [9] that examined children with concurrent PJP and malignancy. The study included very few cases and, even within this limited cohort, no significant difference in outcomes was observed between the two dosing groups. Despite these limitations, the regimen was adopted as the gold standard and has remained so, based primarily on historical practice rather than solid comparative evidence.

No randomized controlled trials have been conducted to date to establish the optimal therapeutic dose of TMP-SMX or to define its minimum inhibitory concentration (MIC). Determining the MIC has proven particularly difficult because *Pneumocystis jirovecii* cannot be cultured with standard microbiological methods, preventing conventional susceptibility testing and pharmacodynamic assessments [10,11]. This limitation complicates efforts to tailor therapy and contributes to the broader challenge of optimizing TMP-SMX dosing in a clinically diverse patient population, as in the absence of culture-based targets or validated pharmacokinetic thresholds, current therapeutic regimens reflect historical consensus rather than precision-guided endpoints.

The absence of an optimal dosing regimen for TMP-SMX in the treatment of PJP is multifactorial. PJP affects a heterogeneous patient population, including those with HIV, cancer, autoimmune diseases, and organ transplants, each characterized by varying immune status, drug tolerances, and disease severity depending on the underlying condition and associated treatments, such as antiretroviral therapy, cytotoxic chemotherapy, corticosteroids, calcineurin inhibitors, biologic agents, and other disease-modifying immunosuppressants [12]. This heterogeneity underscores the need for personalized dosing approaches that incorporate individual factors such as pharmacokinetics and immune phenotype.

Although high-dose TMP-SMX remains the standard approach, its clinical benefit in non-HIV patients is not well established and its toxicity often necessitates early discontinuation, potentially compromising treatment outcomes [13]. Poor tolerance is common in these patients due to factors such as older age, comorbidities, and concurrent immunosuppressive therapies like corticosteroids or calcineurin inhibitors [12]. Adverse effects, including bone marrow suppression, renal dysfunction, and electrolyte disturbances, can force treatment interruption and a switch to second-line agents, which are generally considered less effective [14]. Furthermore, individual variations in pharmacokinetics and host-specific characteristics can influence drug levels and tolerability, adding another layer of complexity to dosing decisions and reinforcing the need for individualized approaches.

The limited availability of large-scale, controlled trials means that dosing guidelines often rely on retrospective data or expert opinion. Furthermore, the higher mortality observed in non-HIV patients may stem from the more rapid disease progression and the dysregulated, exaggerated immune response following treatment initiation, highlighting the need for dosing strategies that account for both pathogen clearance and immune modulation; however, standardized recommendations are currently lacking, making the establishment of a universally optimal regimen challenging [15].

Recent studies have aimed to elucidate optimal dosing strategies. A retrospective cohort study [16] involving 136 hospitalized HIV-negative, immunocompromised patients with PJP compared two different TMP-SMX dosing regimens; 55 patients received an average TMP dose of 8.7 mg/kg/day, while 81 received conventional-dose TMP-SMX, averaging 17.8 mg/kg/day. Although mortality did not significantly differ between the groups, treatment completion was more likely, and adverse effects less frequent, in the low-dose group.

Another four-center retrospective study [13] assessed the efficacy and toxicity of low-dose TMP-SMX in non-HIV-infected patients. Patients were categorized into a conventional-dose group (TMP, 15 to 20 mg/kg/day; 36 patients) and a low-dose group (TMP, <15 mg/kg/day; 41 patients). The survival rates were comparable between the two therapy plans, with the low-dose regimen (mean TMP dose 10.8 mg/kg) being better tolerated, as adverse effects such as vomiting, anorexia, and low platelet counts were more frequently observed in the conventional-dose group (mean TMP dose 17.5 mg/kg).

Additionally, a single-center retrospective observational cohort study from Japan [16] investigated the differences between low-dose and conventional-dose TMP-SMX in non-HIV-infected patients. The low-dose group (n = 24) received TMP at 4–10 mg/kg/day and SMX at 20–50 mg/kg/day, while the conventional-dose group (n = 29) received TMP at 10–20 mg/kg/day and SMX at 50–100 mg/kg/day. The study reported 30- and 180-day survival rates of 69% and 51%, respectively, in the conventional-dose group and 95.8% and 91%, respectively, in the low-dose group [17]. The mortality rate for the low-dose regimen sharply diverged from the reported in-hospital mortality rates for non-HIV PJP, ranging from 30% to 60% [17]. Additionally, adverse effects were also less prevalent in the low-dose group, with reported rates of 58.3% versus 72.4% in the conventional-dose group.

Supporting evidence comes from a retrospective multicenter study [18] that examined outcomes among low-dose (22 patients receiving ≤10 mg/kg/day), intermediate-dose (30 patients receiving 10–15 mg/kg/day), and high-dose (29 patients receiving 15–20 mg/kg/day) TMP-SMX regimens. The study found comparable 30-day survival rates of 100%, 93.3%, and 96.7%, respectively, indicating no significant difference in survival across dosing groups. However, severe adverse reactions were observed less frequently in the low-dose group, reinforcing that dose selection should account not only for efficacy but also for safety when managing non-HIV patients with PJP.

**Figure 1:**
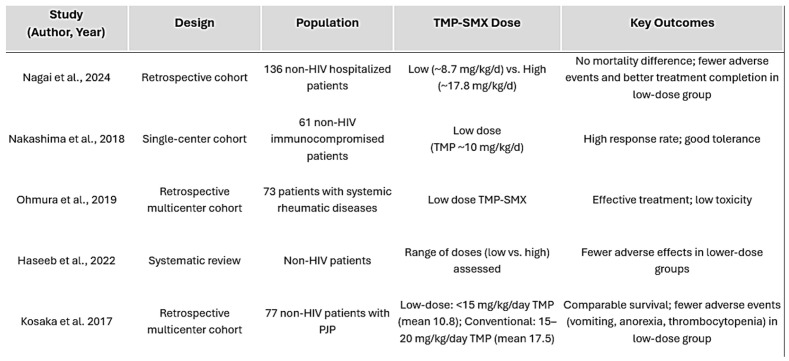
Summary of representative studies evaluating TMP-SMX dosing strategies in non-HIV immunocompromised patients with *Pneumocystis jirovecii* pneumonia. Studies adapted from: Nagai et al. [16], Nakashima et al. [17], Ohmura et al. [18], Haseeb et al. [7], Kosaka et al. [13].

The following table (Figure 1) summarizes the aforementioned key studies assessing low-dose versus high-dose TMP-SMX regimens, including study design, population, dosing approach, and outcomes.

Although these recent studies have suggested that lower-dose TMP-SMX regimens may achieve similar outcomes with fewer adverse effects, the interpretation of the findings requires careful consideration. The retrospective nature of the data, the variability in clinical decision making, and the lack of standardized diagnostic and severity criteria introduce substantial uncertainty. Translating insights from retrospective clinical studies into real-world practice requires caution and a deeper understanding of the interplay between pathogen and host immune responses. A closer examination of these limitations is necessary to better understand how applicable and reliable the current evidence truly is in guiding treatment decisions.

## 4. Methodological Challenges in TMP-SMX Dosing Evidence

Several limitations in the existing studies call for caution when interpreting the apparent benefits of low-dose TMP-SMX in non-HIV PJP. In many cases, higher doses may have been reserved for patients with more severe disease, greater immunosuppression, or multiple comorbidities, all of which independently increase the risk of mortality and may have led clinicians to choose a more aggressive dosing strategy. This introduces potential selection bias, particularly in the absence of randomization. Furthermore, it is rarely clarified how many patients in each dosing group required admission to the intensive care unit. If those receiving lower doses were less critically ill and therefore less likely to require intensive care, any observed mortality benefit may reflect baseline differences in severity rather than true differences in treatment efficacy. The degree of respiratory failure at presentation and prior to intensive care unit (ICU) transfer, as well as objective severity measures such as the Sequential Organ Failure Assessment (SOFA) score, are infrequently reported. Without these data, it is difficult to discern whether any deterioration was due to progressive PJP, secondary sepsis, or complications associated with hospitalization and immunosuppression, including corticosteroid therapy.

Diagnostic certainty presents an additional layer of complexity. Since *Pneumocystis jirovecii* cannot be cultured, diagnosis relies on molecular techniques such as polymerase chain reaction or enzyme-linked immunosorbent assay, which are often performed on non-uniform respiratory specimens including sputum, tracheal aspirates, or bronchoalveolar lavage. This raises the possibility that Pneumocystis genetic material may have been detected in patients without active infection, particularly in immunocompromised hosts who may carry the organism asymptomatically. In some cases, TMP-SMX may have been initiated empirically to provide broad-spectrum coverage and the diagnosis of PJP established or presumed afterward. If a lower dose had already been started, it may have been maintained despite diagnostic uncertainty. These overlapping variables limit our ability to determine whether the observed outcomes with low-dose regimens reflect true efficacy or the influence of diagnostic and clinical ambiguity. Moreover, the rationale for dose selection is often underreported, raising the question of whether low doses were used out of clinical caution, diagnostic uncertainty, or intent to limit toxicity, rather than with confidence in their therapeutic equivalence.

Patients who discontinued TMP-SMX due to adverse effects represent another underexplored subgroup in these studies. Retrospective analyses often fail to specify the timing of discontinuation, the duration of prior treatment, or the patients’ clinical trajectories at the point of treatment interruption. In the absence of data on parameters such as the Horowitz Index for lung function (P/F Ratio) or SOFA scores, it remains unclear whether patients were improving, stable, or deteriorating when TMP-SMX was halted. If patients worsened after stopping treatment, this could suggest a potential therapeutic impact for TMP-SMX, and discontinuation due to toxicity may have directly influenced mortality. Conversely, if the patients were already declining prior to the onset of adverse effects, discontinuation may have been a consequence rather than a cause of clinical deterioration. Furthermore, information is often lacking on what agents were used as second-line therapies, when they were initiated, and whether their use correlated with different clinical outcomes. It also remains unclear whether patients received consistent corticosteroid regimens across groups, or whether some had already been exposed to corticosteroids prior to PJP diagnosis, further confounding the interpretation of both efficacy and adverse effects.

Non-HIV immunocompromised patients with PJP often exhibit a stronger inflammatory reaction following treatment initiation, which may contribute to poorer outcomes. These immunological differences, combined with differences in age, comorbidities, and treatment context, highlight the need to distinguish between HIV-infected and non-HIV-infected populations when evaluating therapeutic approaches and treatment responses.

## 5. HIV vs. Non-HIV Patients with PJP

*Pneumocystis jirovecii* pneumonia (PJP) has long been recognized as a key AIDS-defining illness, with over 90 percent of infections occurring in patients with CD4+ T lymphocyte counts below 200 cells per mm^3^ [19]. The implementation of prophylaxis with TMP-SMX and the early initiation of antiretroviral therapy have significantly reduced the incidence of PJP in HIV-infected individuals. In contrast, there has been a marked increase in PJP cases among non-HIV immunocompromised populations, prompting renewed attention to diagnostic and treatment strategies tailored for this more heterogeneous group.

Non-HIV patients often experience more rapid disease progression and are at higher risk of respiratory failure, intensive care unit admission, and mortality compared to HIV-infected individuals [20,21,22]. While the precise mechanisms behind these differences remain unclear, one proposed explanation is that the absence of the uniform CD4+ T cell suppression seen in HIV allows for a more reactive or less controlled inflammatory response in non-HIV patients, particularly following the initiation of treatment. This hypothesis remains plausible but unproven [23].

Immune dysfunction in non-HIV patients is diverse and depends on the underlying disease and the nature of immunosuppressive therapy. For instance, a cancer patient receiving cytotoxic chemotherapy exhibits a different immunologic profile compared to a patient on long-term corticosteroids, calcineurin inhibitors, or biologic agents. This variability not only increases the risk of opportunistic infections but may also influence the nature and severity of the immune response during treatment, adding further complexity to clinical decision making.

Several overlapping factors may contribute to the worse outcomes seen in non-HIV PJP patients [24,25]: (a) A more intense or dysregulated immune response may lead to extensive lung damage and accelerated disease progression, especially after antimicrobial therapy begins. (b) Delayed diagnosis, often due to atypical symptoms, may result in advanced disease by the time treatment is initiated. (c) The immunosuppressive background varies widely among non-HIV patients, making their responses to infection and therapy less predictable. (d) The absence of universal prophylaxis and inconsistent responses to treatment may further contribute to suboptimal outcomes in this population.

Within this context, the question arises as to whether the standard TMP-SMX dosing regimen is appropriate for non-HIV patients or whether a dose reduction could offer a better balance between efficacy and tolerability. The severity of the immune reaction is influenced by many interrelated factors. These include patient age, comorbidities, organ dysfunction scores such as the SOFA score, intensive care unit status, the timing and reason for therapy discontinuation, and the type and frequency of adverse effects. It is difficult to isolate which of these parameters plays the most significant role in determining clinical outcomes. However, among all these factors, TMP-SMX dosing is one of the few therapeutic decisions directly controlled by the treating physician.

A recent unsupervised cluster analysis of 481 immunocompromised patients with PJP (PRONOCYSTIS cohort) identified three phenotypes. Cluster 1, composed primarily of HIV-positive patients, exhibited the highest survival and lowest SOFA scores. Cluster 2 was enriched for non-HIV individuals with hematologic malignancies and solid tumors and showed significantly worse outcomes. Cluster 3 consisted mainly of patients with solid organ transplants, long-term corticosteroid exposure, and significant respiratory and renal comorbidities, with intermediate prognosis. These phenotypic patterns highlight the prognostic relevance of underlying disease and immune status in guiding treatment decisions and emphasize the importance for personalized, patient-oriented therapeutic approaches in non-HIV PJP [26].

As such, whether a dose reduction can help strike the critical balance between controlling infection and minimizing harmful immune-mediated injury must be rigorously studied.

## 6. Bridging Infection and Autoimmunity

While this review synthesizes clinical and immunological insights, some of the proposed mechanisms remain hypothetical and should be interpreted with caution. Although *Pneumocystis jirovecii* is an opportunistic pathogen, the lung injury observed in PJP may not be directly caused by the microbial burden itself but rather by the host’s exaggerated and poorly regulated immune response [5]. This is particularly evident in non-HIV immunocompromised patients, whose immune systems are not uniformly suppressed as in HIV infection but instead variably altered depending on the underlying condition and immunosuppressive therapy. In these patients, clinical deterioration may follow the initiation of antimicrobial treatment, potentially due to the disintegration of *P. jirovecii* organisms and the release of antigenic fragments, which in turn stimulate a vigorous inflammatory response [24]. This antigen-driven immune activation has been proposed as a possible mechanism of lung injury in PJP, as experimental models and translational immunology studies have speculated that it may contribute to alveolar damage through TNF-α release, neutrophil recruitment, and inflammasome signaling pathways [27,28]. The fungicidal action of TMP-SMX leads to rapid pathogen lysis and antigen release, further intensifying the inflammatory cascade and possibly triggering further lung injury through cytokine surges and inflammasome activation.

This process mirrors autoimmune-like responses, where immune dysregulation drives tissue damage. The post-treatment flare in inflammation observed in PJP resembles phenomena such as the Jarisch–Herxheimer reaction or immune reconstitution inflammatory syndrome, in which the immune system overreacts to microbial antigens following therapy. In HIV-infected individuals, *P. jirovecii*–associated IRIS is infrequently reported under standard treatment, likely due to well-characterized and predictable immune recovery patterns [29]. In contrast, IRIS-like inflammatory flares have been recently reported in immunocompromised non-HIV patients [30], but systematic data are lacking. The diversity of immunosuppressive conditions and therapies in this population complicates the ability to distinguish immune reactivation from infectious progression and makes it unclear to what extent TMP-SMX may function, beyond its antimicrobial action, as a trigger for immune overactivation in different patient subgroups. In this way, the immune pathology of PJP straddles the boundary between infection and autoimmunity without clear mechanistic answers for each individual.

An additional layer of complexity is introduced by the use of corticosteroids. These are frequently administered as adjunctive therapy to dampen inflammation in moderate to severe PJP. Some studies suggest a potential mortality benefit when corticosteroids are given early in the disease course [23,31]. However, their immunosuppressive effect may predispose patients to secondary infections or delay microbial clearance [32], particularly in individuals already receiving long-term steroid therapy or other forms of immunosuppression. Whether corticosteroids ultimately provide more benefit than harm remains uncertain and likely depends on timing, dose, disease severity, and host immune status [33].

This multifaceted immune landscape underscores the need for individualized therapeutic strategies that achieve balance. While antimicrobial efficacy remains a primary objective, mitigating excessive immune activation is equally critical. TMP-SMX dosing, therefore, requires further exploration. Beyond its pharmacological potency, it may serve as a modifiable factor influencing the severity of treatment-induced immune responses. Lowering the dose could potentially reduce not only adverse drug effects, but also the magnitude of downstream inflammatory injury, especially in patients predisposed to hyperinflammation. These considerations argue for a more personalized approach to TMP-SMX therapy, one that integrates individual immune profiles and the risk of treatment-induced inflammation.

To date, direct clinical immunological studies confirming these mechanistic pathways in non-HIV PJP are lacking, and current interpretations are largely extrapolated from observational patterns and related immune conditions. Understanding PJP through this lens helps to bridge infectious disease and autoimmunity. It frames the clinical dilemma not simply as a matter of eradication but of immune calibration. Whether modifying the TMP-SMX dosage can provide a safer and more effective way to achieve this balance remains a clinically important and timely question.

## 7. “Should I Stay or Should I Low?”

The key question in this review is whether an individualized lower dose of TMP-SMX may be more beneficial for select PJP patients, as the optimal dosing remains controversial, with conflicting results reported in various studies [34].

TMP-SMX exhibits important pharmacokinetic and pharmacodynamic properties that enhance its clinical efficacy. The drug is well absorbed orally, achieving peak plasma concentrations within 1 to 4 h. TMP has a volume of distribution of approximately 0.6 L/kg and is 44% to 69% protein bound, while SMX has a volume of about 1.5 L/kg with 70% to 90% protein binding. TMP is lipophilic, aiding in tissue penetration, while SMX is more hydrophilic. Therapeutic blood concentrations typically range from 1 to 10 µg/mL for trimethoprim and 10 to 50 µg/mL for sulfamethoxazole, with elimination primarily occurring via the kidneys and half-lives of 8 to 10 h for trimethoprim and 6 to 12 h for sulfamethoxazole [35,36]. While these levels are considered therapeutic for most bacterial infections, their applicability to *Pneumocystis jirovecii* pneumonia remains unclear, as their pharmacodynamic relevance in this context has yet to be clarified.

While the bactericidal effect of TMP-SMX stems from the sequential inhibition of folate synthesis, a major limitation in *Pneumocystis jirovecii* pneumonia is the absence of an established minimum inhibitory concentration (MIC), largely due to the organism’s resistance to standard culturing methods. The MIC, defined as the lowest antibiotic concentration that inhibits visible microbial growth, is central to determining clinical breakpoints. According to the European Committee on Antimicrobial Susceptibility Testing (EUCAST), these breakpoints are updated annually to guide antimicrobial selection and define susceptibility thresholds [11]. Despite a persistent lack of MIC data, low-dose regimens of TMP-SMX have demonstrated adequate efficacy for *Pneumocystis jirovecii* pneumonia (PJP) prevention. This may be attributed to an inferred low MIC, as prophylactic doses (approximately TMP 1.3 mg/kg/day) are frequently adequate for preventing PJP [37]. Notably, the recommended prophylactic dose typically aligns with the treatment dose required to achieve sufficient serum and tissue concentrations that exceed the MIC for many bacterial infections [38,39,40]. This suggests that lower therapeutic doses might also be effective in treating active *Pneumocystis jirovecii* pneumonia, particularly in patients with less severe immunosuppression, where the host immune response may still contribute significantly to pathogen clearance.

Immunomodulation could be another critical factor in PJP management. Recent studies highlight the role of immune cells in PJP pathogenesis, suggesting that targeting these cells could enhance patient outcomes [41,42]. In many cases, lung damage and poor outcomes are more of a result of an excessive inflammatory response than the infection itself. While TMP-SMX targets the pathogen directly, immune modulation aims to reduce the collateral damage caused by the host’s enhanced inflammatory response, which may intensify even more following pathogen lysis induced by antimicrobial therapy. Corticosteroids are commonly used for this purpose, though newer approaches involving cytokine modulation and other immunotherapies are being explored [43,44]. These strategies aim to dampen the excessive inflammatory response and, when combined with antimicrobial therapy, may improve outcomes in severe PJP cases.

Emerging immunotherapeutic approaches, such as cytokine modulation, aim to precisely attenuate the hyperinflammatory response without inducing systemic immunosuppression. Theoretically, pathway-specific blockers (e.g., IL-6, IL-1 inhibitors) could modulate the damaging immune response following pathogen lysis while preserving essential host defenses. However, such strategies require further validation, as they may also increase vulnerability to secondary infections. Tailoring immunotherapy to individual inflammatory profiles might help achieve a more favorable risk–benefit balance in PJP management [41,43].

Additionally, non-HIV immune reconstitution inflammatory syndrome (non-HIV IRIS) may be triggered during recovery in immunocompromised patients, as the infection improves while the immune system is being restored. It is an inflammatory disorder caused by antigens, drugs or microorganisms present prior to immune recovery, hence, fluctuations in the immune environment may intensify it [45]. Uncontrolled immune activation, potentially driven by inflammasome dysregulation following antimicrobial-induced pathogen lysis, may lead to clinical deterioration even after initial improvement [46]. This raises the possibility of a dose-dependent effect, where a lower TMP-SMX dose could mitigate excessive immune stimulation. In this context, adjusting the antimicrobial dose itself may serve as a form of immunomodulation, potentially offering an alternative or complement to adjunctive corticosteroids [20,47].

It remains unclear whether lower TMP-SMX dosing alone could mitigate the immune response sufficiently to eliminate the need for corticosteroids or whether the combination is synergistic [33]. Furthermore, PJP itself may induce transient immunoparalysis due to overwhelming immune activation, a process potentially exacerbated by adjunctive corticosteroids. A recent study evaluating TMP-SMX without corticosteroids in select non-HIV patients suggested favorable outcomes in mild cases, though this remains unproven for moderate-to-severe disease [48].

The role of corticosteroids as adjunctive therapy in PJP remains debated, with no randomized trial to provide definitive guidance. Some retrospective studies suggest improved survival in patients on corticosteroid therapy, while others report no significant difference in mortality rates [48,49,50]. There is also a study associating corticosteroid use with a faster occurrence of death (time ratio: 2.48; 95% CI 1.01–6.08; *p* = 0.048) [49]. These conflicting findings may reflect variability in corticosteroid regimens (e.g., pulse versus standard dosing), differences in total corticosteroid exposure, timing of administration, and the risk of secondary infections in critically ill patients. Patients in intensive care units or under mechanical ventilation may be particularly vulnerable to corticosteroid-related complications, further complicating interpretation.

Critically ill patients with PJP admitted to the ICU, especially those requiring mechanical ventilation, represent a distinct subgroup. Their altered physiology, including increased volume of distribution due to fluid shifts and hypoalbuminemia, may affect TMP-SMX pharmacokinetics and necessitate adjusted dosing. Moreover, these patients are at heightened risk of multidrug-resistant secondary infections, which may surpass the immediate threat posed by Pneumocystis itself. Sepsis-induced immunoparesis, malnutrition, and renal losses of immunoglobulins can further compromise host defenses, complicating therapeutic decisions [51,52].

As clinical deterioration may be expected during the initial phase of PJP treatment, the missing link to explain the efficacy and potentially favorable outcome of the lower TMP-SMX dose could be hidden in the non-HIV lung hyperinflammation induced by the bactericidal effect of the treatment itself and is probably dose dependent. Consequently, the chemotherapeutic dogma “hit hard, hit fast” may not be applicable for PJP. Deterioration is probably due to an exaggerated inflammatory response caused by antimicrobial-induced microorganism lysis in the lung. Steroids may alleviate the response in severe PJP, exactly as in severe COVID-19 pneumonia, pneumococcal/tuberculous meningitis, Jarisch–Herxheimer reaction, or IRIS [47].

**Figure 2:**
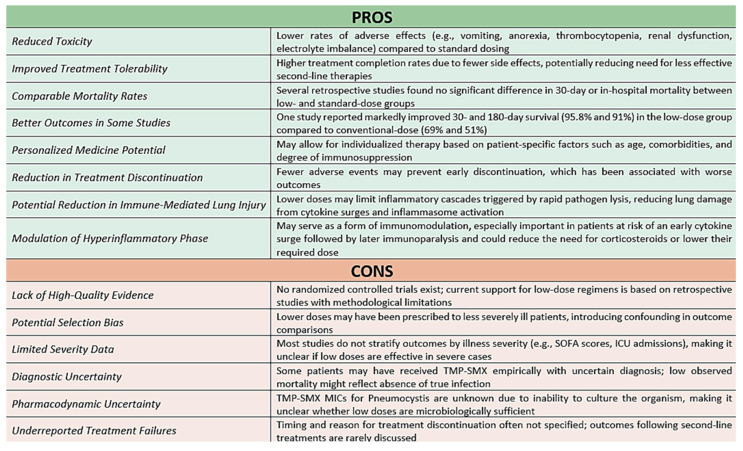
Benefits and risks associated with individualized lower-dose TMP-SMX regimens in non-HIV immunocompromised patients with *Pneumocystis jirovecii* pneumonia.

Non-HIV PJP may involve a biphasic immune response. Initially, antimicrobial-induced pathogen lysis can provoke a surge of cytokines and inflammation. However, this can be followed by an immunoparalytic phase characterized by T-cell exhaustion and susceptibility to secondary infections—particularly in the context of corticosteroid use. Recognizing this dynamic may help refine the timing and intensity of immunomodulatory therapies aiming to mitigate early hyperinflammation without prolonging immunosuppression [3]. Therefore, a lower TMP-SMX dose could ameliorate the pulmonary hyperinflammatory reaction, allowing for homeostasis and thus preventing further exacerbation of the inflammatory milieu [8]. A balanced view of the key advantages and limitations of individualized low-dose TMP-SMX strategies is summarized below (Figure 2).

While the current evidence is insufficient to establish definitive guidelines, clinicians may cautiously consider individualized, lower-dose TMP-SMX strategies in select non-HIV PJP patients, particularly those with mild-to-moderate disease or at high risk for drug toxicity. However, the variability in study quality, patient populations, and outcome definitions leaves key questions unanswered. Prospective, pragmatic randomized trials, ideally incorporating inflammatory biomarkers and stratified by host immune profiles, are needed to define optimal dosing strategies and clarify the role of individualized treatment in this population.

## 8. Conclusions

The optimal dosing strategy for TMP-SMX in non-HIV patients with PJP remains an area of active debate. While high-dose regimens have traditionally been considered the standard of care, emerging evidence raises the hypothesis of the clinical efficacy of lower doses that may reduce toxicity without compromising outcomes. Recent data suggest that individualized, lower-dose TMP-SMX regimens may improve tolerability without compromising efficacy in non-HIV immunocompromised patients, potentially by achieving a more favorable balance between antimicrobial activity and immunological control. However, these findings are primarily based on retrospective and observational studies and require confirmation through randomized controlled trials with well-defined, homogeneous non-HIV immunocompromised populations. Further randomized studies should directly compare different TMP-SMX doses in non-HIV PJP, incorporating not only clinical endpoints but also biomarkers of inflammation and host response to optimize efficacy, tolerability, and immune-related outcomes. In the interim, clinicians may cautiously consider individualized, lower-dose strategies in selected high-risk patients, while remaining guided by clinical judgment and evolving evidence.

## Data Availability

Not applicable.

## Abbreviations

The following abbreviations are used in this manuscript:

AIDS	Acquired Immune Deficiency Syndrome
HIV	Human Immunodeficiency Virus
IRIS	Immune reconstitution inflammatory syndrome
ICU	Intensive care unit
MIC	Minimum inhibitory concentration
PJP	*Pneumocystis jirovecii* pneumonia
SOFA Score	Sequential Organ Failure Assessment Score
TMP-SMX	Trimethoprim-sulfamethoxazole
